# Dietary Selenium Source and Level Influence Growth Performance, Blood Parameters, Meat Quality, and Selenium Deposition in Broilers

**DOI:** 10.3390/ani16182821

**Published:** 2026-09-08

**Authors:** Xingbo Liu, Ziqi Zhao, Jianmin Yuan, Lei Yan, Wei Nie

**Affiliations:** 1 College of Animal Science and Technology, Fujian Vocational College of Agriculture, Fuzhou 360119, China; liuxingbo1997@outlook.com; 2 State Key Laboratory of Animal Nutrition, College of Animal Science and Technology, China Agricultural University, Beijing 100193, China; 18303444433@163.com (Z.Z.); yuanjm@cau.edu.cn (J.Y.); 3 Shandong New Hope Liuhe Group Co., Ltd., Qingdao 266000, China; yanlei@newhope.cn

**Keywords:** nano-selenium, selenium source, antioxidant capacity, selenium deposition, performance, broiler

## Abstract

This study investigated the effects of dietary nano-selenium (nano-Se) levels and Se sources on growth performance, blood parameters, meat quality, and tissue Se deposition in broilers. In Experiment 1, 480 broilers were assigned to five treatments (0, 0.10, 0.20, 0.30, and 0.40 mg/kg nano-Se) to assess the dose-dependent effects of nano-Se, with 0.20–0.22 mg/kg identified as the optimal dose through quadratic regression. In Experiment 2, 480 broilers received either a control diet or diets supplemented with 0.20 mg/kg Se from nano-Se, sodium selenite (SS), or two organic Se sources (Se-enriched yeast 1 and 2, SY1 and SY2). Supplementation with 0.20–0.30 mg/kg nano-Se improved (*p* < 0.05) antioxidant status, carcass yield, muscle redness at 45 min post-slaughter, and Se concentration in serum. At 0.20 mg/kg, nano-Se, SY1, and SY2 showed greater improvements in carcass traits, and antioxidant status than SS (*p* < 0.05), with comparable efficacy between nano-Se and organic sources. Nano-Se, organic Se sources, and SS increased Se deposition in serum and breast muscle compared to the control (*p* < 0.05). In conclusion, 0.20 mg/kg nano-Se is recommended for broiler diets, demonstrating superior bioefficacy to inorganic Se and comparable effects to organic Se sources.

## Simple Summary

Selenium is an essential trace element crucial for the normal growth, development, and overall health of animals. This study investigated nano-selenium synthesized by the *Bacillus subtilis S12* strain, which forms exceptionally small particles that may be more readily absorbed by poultry. We tested different amounts of nano-selenium and compared it with traditional selenium sources, including a chemical form and two yeast-based forms. Results showed that adding an appropriate amount of nano-selenium improved the chickens’ antioxidant capacity, increased meat yield, and enhanced meat color. It also increased the amount of selenium stored in the body, which is important for nutritional value. Nano-selenium performed as well as or better than the traditional organic selenium sources and clearly outperformed the chemical form. These findings offer practical guidance for improving selenium supplementation strategies in broiler production.

## 1. Introduction

Selenium (Se) is an essential trace element crucial for the normal growth, development, and overall health of animals. It exerts a wide range of biological effects, including enhancing immunity, antioxidant capacity, growth performance, meat quality, reproductive performance, and egg quality [1,2,3,4]. Se’s unique role as the 21st amino acid, selenocysteine (Sec), differentiates it from other nutritional elements. It is directly incorporated into proteins, where it plays critical roles in regulating redox homeostasis through selenoproteins [5].

Two forms of Se are commonly used in animal nutrition: organic Se, typically in the form of selenomethionine, and inorganic Se, such as selenite and selenate [6]. While inorganic Se is cost-effective, organic Se is generally more bioavailable and demonstrates a higher retention rate in tissues. Organic Se, particularly Se-enriched yeast (SY), has been shown to offer superior antioxidant and immune-enhancing properties, with less toxicity compared to inorganic Se [7,8]. Organic Se is absorbed actively in the gastrointestinal tract and converted into hydrogen selenide, which is utilized for selenoprotein synthesis [9].

Nanotechnology has brought significant advancements in the form of Se supplementation, driving research in the field of animal nutrition. Nanoparticles possess higher physical activity and chemical neutrality [10]. Studies show that nano-selenium (nano-Se) are absorbed more efficiently in the intestine and retained less in detoxifying tissues than sodium selenite (SS) and selenomethionine (SM) [11].

However, the effects of nano-Se on broiler growth performance have been inconsistent. Elkhateeb et al. [12] reported that dietary supplementation with 0.20 and 0.30 mg/kg nano-Se improved body weight at 35 d and average daily gain during days 1–35 in broilers, compared with the control group. Similarly, Ali et al. [13] found that dietary supplementation of 0.30 mg/kg nano-Se significantly increased body weight at 42 d and reduced the feed conversion ratio in broilers. In contrast, Bakhshalinejad et al. [1] reported no significant effect on the growth performance of broilers from day 1 to day 42 when supplemented with 0.10 mg/kg or 0.30 mg/kg of SS, SY, or nano-Se. Chen et al. [14] similarly observed that dietary supplementation with 0.50 mg/kg of SS, SY, SM, or nano-Se had no significant effect on the growth performance of broilers at 56 d. The inconsistencies in findings could be attributed to differences in experimental conditions, Se dosages, rearing periods, and broiler breeds across studies. Consequently, the exact impact of nano-Se on broiler growth performance remains unclear, highlighting the need for further research to explore its mechanisms and determine optimal dosages.

Furthermore, the synthesis method of nano-Se may critically influence its bioactivity. Conventional physical and chemical synthesis methods are often energy-intensive and may generate harmful by-products [15]. In contrast, biological approaches using bacteria [16,17], fungi [18], and plant extracts [19] have emerged as safer and more cost-effective alternatives [15]. This study utilized nano-Se synthesized by *Bacillus subtilis S12*. However, the effects of biologically synthesized nano-Se on broiler growth performance, carcass traits, and Se retention—especially in comparison with traditional Se sources—remain largely unexplored.

Our previous research found that supplementation with 0.20 mg/kg nano-Se alleviated intestinal oxidative stress and inflammation, promoting the development of intestinal goblet cells in 21-day-old broilers [20,21]. However, whether this protective effect translates into improved overall growth performance and whether a dose–response relationship exists remain unknown. Therefore, the first objective of this study was to assess the dose-dependent effects of nano-Se on growth performance, carcass traits, and Se retention in tissues. The second objective was to compare the effects of nano-Se with those of traditional organic and inorganic Se sources to better understand the relative benefits of each form in broiler diets.

## 2. Materials and Methods

### 2.1. Animal Care

The research protocol was reviewed and approved by the Animal Ethics Committee of China Agricultural University, Beijing, China (CAU-20210522), and all experimental procedures were conducted in accordance with the guidelines for animal experiments set by the National Institute of Animal Health, China.

### 2.2. Management and Housing

#### 2.2.1. Experiment 1

A total of 480 one-day-old Arbor Acres broilers (equal numbers of males and females) were obtained from a local hatchery. The trial was conducted at the Zhuozhou Poultry Experimental Farm of China Agricultural University. The temperature was 33 °C for the first week, then decreased to 23 °C at a rate of 1 degree every two days. Feed and water were supplied ad libitum throughout the experiment.

#### 2.2.2. Experiment 2

A total of 480 one-day-old Arbor Acres broilers (equal numbers of males and females) were obtained from a local hatchery. Husbandry, housing, environmental conditions, and sample collection and analysis were identical to those used in Experiment 1.

### 2.3. Birds, Diets, and Experimental Design

In Experiment 1, broilers were randomly divided into 5 treatments with 6 replicates each and 16 chickens per replicate, with males and females equally represented. The supplemental levels of nano-Se in the diet were 0 (control group), 0.10, 0.20, 0.30, and 0.40 mg/kg of selenium.

In Experiment 2, broilers were randomly divided into 5 treatments with 6 replicates each and 16 chickens per replicate, with males and females equally represented. The control (Con) group was fed with the basal diet; while the nano-Se, sodium selenite (SS), SY1, and SY2 groups received 0.20 mg/kg selenium (based on the selenium content in the diet). The selenium sources were as follows: nano-Se (3000 mg/kg; particle size: 170 nm) was obtained from Beijing Wahmix Bio-technology Co., Ltd. (Beijing, China); sodium selenite (SS) (10,000 mg/kg) was obtained from Jinrunmufeng Co., Ltd. (Beijing, China); SY1 (2000 mg/kg) was obtained from Angel Yeast Co., Ltd. (Beijing, China); and SY2 (2000 mg/kg) was obtained from Lallemand Co., Ltd. (Montreal, QC, Canada). Each treatment group was supplemented with the respective selenium source to achieve 0.20 mg/kg of selenium, based on the Con group.

In the two experiments, the ingredients, diets and feeding regimes were identical. Low-Se corn and Se-deficient trace elements were selected to formulate the diets in this experiment. All diets were formulated to meet or exceed the Feeding Standard of Chicken, China (NY/T 33-2004) (China, 2004) [22]. The composition of the diets and nutrient levels are shown in Table 1. The analyzed Se contents in experimental diets are summarized in Table 2.

### 2.4. Sample Collection and Analysis

#### 2.4.1. Growth Performance

Body weight (BW) and feed intake were recorded for each cage on days 1, 21 and 40, and mortality was recorded daily. Average daily gain (ADG), average daily feed intake (ADFI), and feed conversion rate (FCR) were calculated for all stages of growth (1–21 d, 22–40 d, 1–40 d).

#### 2.4.2. Carcass Traits

On day 40, one male broiler (body weight close to the average weight per cage) per replicate was selected, deprived of feed for 12 h, weighed, and then slaughtered. Carcass parameters were measured and calculated according to the poultry production performance noun terms and metric statistics method (NY/T 823-2004) (China, 2004) [23]. Relative weight was determined for carcass yield (including organs), eviscerated carcass, half-eviscerated carcass, abdominal fat, breast muscle, and thigh muscle. Carcass, eviscerated carcass and half-eviscerated carcass yields were expressed as a percentage of live BW. Abdominal fat, breast muscle, and thigh muscle yields were expressed as a percentage of the eviscerated weight.

#### 2.4.3. Sample Collection

On days 21 and 40, one male broiler, closest to the average weight, was randomly selected from each replicate. Blood samples (4 mL were collected from wing veins, centrifuged at 3000 rpm for 10 min to separate the serum, and then kept at −20 °C for subsequent analysis. Blood samples (4 mL) were collected from the wing vein of the selected broiler, then centrifuged at 3000 rpm for 10 min to separate the serum. The serum was then stored at −20 °C for subsequent analysis. On day 40, the selected broilers were sacrificed by jugular bleeding. The left pectoralis major muscle was used to determine drip loss, meat color, and pH. Additionally, left breast muscle samples were collected and stored at −80 °C for subsequent analysis of Se content. The right pectoralis major muscle was used to measure cooking loss and shear force.

#### 2.4.4. Meat Quality

pH. The pH values were measured at 45 min and 24 h (the muscle storage for 24 h at 4 °C) after slaughter using a digital pH meter (Model Testo 205, Testo Inc., Sparta, NJ, USA). The pH values were obtained from three different sections of each muscle sample and then the average value was determined.

Meat color. Meat color (including lightness (L), redness (a), and yellowness (b)) was obtained at 45 min and 24 h after slaughter using a fully automatic chromameter (3nh NR-10QC; Shenzhen Three nh Technology Co., Ltd., Shenzhen, China). Each muscle sample was measured 3 times on the dorsal side of the intact skinned pectoralis major muscle, and the average value was taken.

Drip loss. Approximately 15 g of pectoralis major muscle was weighed (M1) and placed in a sealed polyethylene bag. After 24 h at 4 °C, the muscle was reweighed (M2). The drip loss was calculated as: Drip loss (%) = (M1 (g) − M2 (g))/M2 (g) × 100.

Cooking loss. A cuboid sample (1.5 cm × 1.5 cm × 6 cm) was taken on the dorsal side of the intact skinned pectoralis major muscle for the determination of cooking loss. Samples were placed in sealed polyethylene bags in a 75 °C water bath and heated to a central meat temperature of 75 °C (approximately 30 min), removed and cooled to room temperature. Cooking loss was calculated as: Cooking loss (%) = (initial weight (g) − final weight (g))/initial weight (g) × 100.

Shear force. Meat samples after cooking loss were cut into cuboid strips (1 cm × 1 cm × 5 cm) to determine shear force. Each sample was measured six times using a TMS-touch texture analyzer (Food Technology Co., Sterling, VA, USA), and the average value was taken.

#### 2.4.5. Serum Parameters

Serum total cholesterol (TC), triglycerides (TG), lipase (LPS), free fatty acids (FFA), high-density lipoprotein (HDL), low-density lipoprotein (LDL), very-low-density lipoprotein (VLDL), blood urea nitrogen (BUN), total protein (TP), globulin (GLB) and albumin (ALB) were determined using an automatic analyzer and commercial kits from Nanjing Jiancheng Bioengineering Institute (Nanjing, China). The concentrations of malondialdehyde (MDA), glutathione peroxidase (GSH-Px), reduced glutathione (GSH), total antioxidant capacity (T-AOC) were determined using a commercial kits (Nanjing Jiancheng Bioengineering institute, Nanjing, China) following the manufacturer’s instructions.

#### 2.4.6. Se Content Assay

Diets, serum, and left breast muscle samples were pretreated, and Se content was determined according to the method described by Liu et al. [24] with slight modifications. Briefly, samples (0.5–1 g for diets and tissues, or 0.5–1 mL for serum) were digested in a mixture of concentrated nitric acid and perchloric acid (HNO_3_:HClO_4_ = 2:1, *v*/*v*) at 200 °C for approximately 2 h until white fumes appeared. Subsequently, 5 mL of hydrochloric acid solution (HCl:H_2_O = 1:4, *v*/*v*) was added and heated again until white fumes were observed. After cooling, 20 mL ethylenediaminetetraacetic acid (EDTA) solution was added, and the pH was adjusted to 1.5–2.0. Then, 3 mL of 2,3-diaminonaphthalene was added, and the mixture was heated in a boiling water bath for 5 min. After cooling, 4 mL of cyclohexane was added and the mixture was shaken for 8 min. The supernatant was collected, and the Se concentration was determined using a fluorescence spectrophotometer.

### 2.5. Statistical Analysis

All data were analyzed using SPSS 22.0 (SPSS Inc., Chicago, IL, USA). Data were subjected to one-way ANOVA. When a significant effect was detected (*p* < 0.05), means were compared using Duncan’s multiple range test. Orthogonal polynomial contrasts were used to assess the linear and quadratic effects of different nano-Se levels. Quadratic regression analysis was conducted to evaluate the relationship between Se supplementation levels and the response variables, and the vertex of the regression equation was calculated to determine the optimal selenium level. Results are presented as means ± pooled SEM. Differences between means and interactions with *p* < 0.05 were considered statistically significant.

## 3. Results

### 3.1. Experiment 1

#### 3.1.1. Growth Performance

As shown in Table 3, dietary treatment had no effect (*p* > 0.05) on BW, ADG, ADFI, FCR, and mortality rate during the starter (d 1–21), grower (d 22–40), and whole periods (d 1–40) based on ANOVA. However, linear contrasts revealed that ADG during d 22–40 and ADFI during d 1–40 increased with elevated nano-Se levels (*p* < 0.05). Additionally, FCR during d 1–21 was quadratically related to dietary nano-Se levels (*p* = 0.044), with the lowest FCR observed in the 0.20 mg/kg nano-Se group.

#### 3.1.2. Serum Biochemical Parameters

On d 21, supplementing nano-Se, irrespective of its level, linearly and quadratically increased the LPS concentration (*p* < 0.05; Table 4), with the highest values observed in the 0.30 mg/kg nano-Se group. The TC content in the serum of broilers increased linearly with the increase in nano-Se supplementation levels (*p* < 0.05). Broilers fed the basal diet with 0.20 mg/kg nano-Se showed improved TG concentration compared with the Con group (*p* < 0.05).

On d 40, nano-Se addition linearly increased ALB, TG, LDL, VLDL, FFA, LPS concentrations in the serum of broilers, but linearly decreased the HDL concentration (*p* < 0.05). Compared with the Con group, dietary supplementation with 0.30 mg/kg nano-Se significantly increased the TP, ALB, TC, TG, LDL, VLDL, BUN, and LPS levels in the serum (*p* < 0.05).

#### 3.1.3. Serum Antioxidant Status

On d 21, dietary nano-Se supplementation quadratically reduced serum MDA concentration (*p* < 0.001) and quadratically increased serum GSH-Px activity and GSH concentration (*p* < 0.001), with the 0.20 and 0.30 mg/kg groups showing lower MDA and higher GSH-Px and GSH levels than the Con group (*p* < 0.05; Table 5). Serum T-AOC concentration also showed a quadratic response to nano-Se levels (*p* = 0.008). On d 40, the serum MDA level of broilers in the 0.20 mg/kg nano-Se group was significantly lower than that of broilers in the Con group (*p* < 0.05). The addition of nano-Se resulted in quadratic effects (*p* < 0.05) on serum T-AOC, and GSH concentrations, and the serum GSH level of broilers in the 0.40 mg/kg nano-Se group was significantly lower than that in the Con group (*p* < 0.05). Notably, the highest T-AOC, GSH-Px, and GSH levels were observed in the 0.20 mg/kg nano-Se group.

#### 3.1.4. Carcass Traits

Table 6 displayed that dietary treatments had no effect on half-eviscerated carcass yield, eviscerated carcass yield, thigh muscle yield, and abdominal fat yield (*p* > 0.05). However, carcass yield responded quadratically to nano-Se supplementation (*p* = 0.003), with the 0.20 mg/kg nano-Se group showing higher yield than the Con group (*p* < 0.05). Additionally, breast muscle yield exhibited a linear increasing trend with elevated nano-Se levels (*p* = 0.049).

#### 3.1.5. Meat Quality

In Table 7, no significant differences among treatments were observed for pH_45min_, pH_24h_, drip loss, cooking loss, shear force, L_45min_, L_24h_, a_24h_, and b_24h_ (*p* > 0.05). However, dietary nano-Se supplementation linearly increased a_45min_ (*p* = 0.006). The b_45min_ value showed a quadratic response to nano-Se supplementation, with the lowest value observed in the 0.20 mg/kg nano-Se group (*p* = 0.011).

#### 3.1.6. Tissue Se Content

Table 8 indicated that dietary nano-Se supplementation linearly increased serum (*p* < 0.001) and breast (*p* = 0.020) Se content. Additionally, there were no significant differences in serum Se content when the Se addition ranged from 0.10 to 0.40 mg/kg (*p* > 0.05). The treatment supplemented with 0.40 mg/kg nano-Se showed the highest (*p* < 0.05) breast Se content, but there were no significant differences between other treatment groups (*p* > 0.05).

#### 3.1.7. Dietary Se Requirements

The dietary Se requirements of broilers, estimated by the quadratic regression analysis, are presented in Table 9. Based on fitted quadratic regression analyses of FCR from d 1–21, carcass yield, and the activities of GSH-Px, T-AOC, and GSH as well as MDA content at d 21, the optimal dietary Se level was estimated to range from 0.20 to 0.22 mg/kg for broilers fed nano-Se-supplemented diets. Notably, the estimated optimal Se requirement based on GSH-Px activity at 21 d (0.21 mg/kg) was highly reliable (R^2^ = 0.9698). In contrast, antioxidant parameters at 40 d of age showed weaker model fits (R^2^ < 0.38), yielding lower estimated requirements (0.14–0.18 mg/kg). Collectively, the robust quadratic regression analyses (R^2^ > 0.80) consistently indicated that the optimal dietary nano-Se supplementation level ranged from 0.20 to 0.22 mg/kg.

### 3.2. Experiment 2

#### 3.2.1. Growth Performance

The effect of the Se source on the growth performance of broilers is shown in Table 10. No differences among treatments were observed for BW, ADFI, and ADG during d 1–21 (*p* > 0.05). During d 22–40, the SY2 group had a higher ADFI than the SS group (*p* < 0.05), with no differences among the Con, nano-Se, SY1, and SY2 groups. During d 1–40, ADFI was higher in the SY2 and SY1 groups than in the SS group (*p* < 0.05), but did not differ among the Con, nano-Se, SY1, and SY2. No significant differences were observed in FCR or the mortality rate during any period (*p* > 0.05).

#### 3.2.2. Serum Biochemical Parameters

As presented in Table 11, serum ALB, TC and HDL levels were increased (*p* < 0.05) in the nano-Se, SS, SY1, and SY2 groups at day 21 compared to the Con group, but there were no differences between the four groups. Serum TP, GLB and BUN levels in the SS group were significantly higher (*p* < 0.05) than those in the Con and SY2 groups on day 21, while remaining similar to those in the nano-Se group. Regarding TG, the serum TG level was increased in the nano-Se group at day 21 as compared to the other treatment groups. On d 21, the highest (*p* < 0.05) serum levels of LDL, VLDL and LPS were found in the SY1 group, which did not differ from the SS and SY2 groups, while these were lowest in the Con group. On d 40, dietary treatment had no effect on serum biochemical parameters (*p* > 0.05; Table 6), including serum contents of TP, ALB, HDL, and FFA. Compared with other groups, GLB content was highest in the Con group and TC content was lowest in the nano-Se group (*p* < 0.05). Serum TG, VLDL, BUN, and LPS levels were increased (*p* < 0.05) in the SY2 group as compared to the other treatment groups.

#### 3.2.3. Serum Antioxidant Status

The effect of the Se source on the serum antioxidant parameters of broilers is shown in Table 12. Serum MDA levels were decreased (*p* < 0.05) and GSH-Px was increased (*p* < 0.05) at d 21 and 40 in the nano-Se, SY1 and SY2 groups compared to the Con and SS groups. Regarding T-AOC, the highest (*p* < 0.05) T-AOC level was found in the SY1 group at d 21 and 40, which was similar to the SY2 group, while the lowest level was found in the Con group. The GSH level in the serum at d 21 was significantly higher in the nano-Se, SY1, and SY2 groups than in the Con and SS groups (*p* < 0.05).

#### 3.2.4. Carcass Traits

Carcass yield was highest in the nano-Se group and lowest in the SS group (*p* < 0.05; Table 13). Half-eviscerated carcass and eviscerated carcass were lower in the SS group as compared to the other groups (*p* < 0.05). Regarding abdominal fat yield, the highest value was found in the nano-Se group, which was similar to the Con, and SY1 groups, while the lowest value was found in the SY2 group (*p* < 0.05). Additionally, dietary treatment had no effect (*p* > 0.05) on breast muscle yield or thigh muscle yield.

#### 3.2.5. Meat Quality

As presented in Table 14, no significant differences were observed for pH_45min_, pH_24h_, shear force, L_45min_, b_45min_, L_24h_, a_24h_, and b_24h_ for each group (*p* > 0.05). Drip loss was highest in the SS group, which was significantly higher than the other groups (*p* < 0.05). Cooking loss was higher in the SS and SY1 groups than in the nano-Se and SY2 groups (*p* < 0.05). The highest a_45min_ was observed in the SY1 group, which was similar to the nano-Se and SS groups, while the lowest a_45min_ was observed in the Con and SY2 group (*p* < 0.05).

#### 3.2.6. Tissue Se Content

As shown in Table 15, serum Se level was significantly higher in the nano-Se, SS, SY1, and SY2 groups than in the Con group (*p* = 0.001), with no differences among these four groups. Breast Se content was significantly higher in the nano-Se, SY1, and SY2 groups than in the Con group (*p* = 0.010), and did not differ from that of the SS group.

## 4. Discussion

### 4.1. Growth Performance

Se, an essential trace element, is crucial for animal growth and development, particularly in supporting antioxidant defenses, thyroid hormone regulation, and immune function. Several studies have shown that nano-Se can positively influence broiler growth performance [12,13]. However, conflicting evidence exists, as other research has found no significant effects of nano-Se on broiler growth performance [1,14]. In the present study, while dietary nano-Se did not significantly affect overall broiler growth performance based on ANOVA, linear contrast analysis revealed a significant increase in ADG during the grower phase (days 22–40) as Se levels increased. This finding suggests a potential role for nano-Se in supporting growth during the grower phase. The observed trend may be attributed to the role of Se in antioxidant defense. As a key component of GSH-Px, Se enhances intracellular antioxidant capacity, thereby mitigating oxidative stress during periods of high metabolic demand [12]. However, whether this antioxidant effect drives growth performance improvement requires further investigation.

The bioavailability of nano-Se may vary between studies, possibly due to differences in particle size and synthesis methods. Nanoparticle size plays a crucial role in absorption efficiency in the gut, affecting their distribution and function within the body [2,25]. Research suggests that smaller nanoparticles are more capable of penetrating cell membranes and participating in metabolic processes, leading to greater bioavailability [10]. This variability may explain why some studies report more significant effects of nano-Se, while others observe no such growth benefits. Additionally, Se levels in the basal diet were different in the Con groups of these studies, which could have influenced the outcomes. Se levels and sources in breeder diets can also affect the growth performance of offspring [26]. In our trial, there was no nutritional deficiency in broilers at hatching, and Se levels of 0.08–0.10 mg/kg appeared to maintain broiler growth performance under our study conditions. This suggests that under conditions where baseline nutrition is adequate, additional nano-Se supplementation may not provide significant growth benefits.

Based on the quadratic regression analysis of FCR, the estimated optimal dose of Se for growth performance was approximately 0.20 mg/kg. To further explore the effects of this optimal level, we compared the impacts of different Se sources (nano-Se, SY, and SS) at this dose. The results of this study indicated that nano-Se and SY supplementation increased the ADFI in broilers compared to SS group. Adequate feed intake is essential for improving animal survival and growth potential. Nano-Se may stimulate feed intake through mechanisms like enhancing digestive efficiency and modulating gut microbiota balance [12,13]. However, the precise mechanisms still require further validation. Additionally, supplementing the diet with 0.20 mg/kg of nano-Se and SY did not significantly affect the growth performance of broilers. These results align with the findings of Li et al. [27], who reported that the growth performance of broilers at day 90 following supplementation with 0.30 mg/kg of SY, Met-Se, or nano-selenium was similar to that observed with supplementation of 0.30 mg/kg of SS. However, Ibrahim et al. [28] observed that adding the same levels of Met-Se and nano-Se to the diet significantly improved broiler growth performance at 38 days, compared to the SS group receiving 0.30 and 0.60 mg/kg.

These discrepancies may be attributed to variations in the sources, dosages, and feeding conditions of Se, indicating that both the form and dosage of Se significantly influence its biological effects. Future research should further explore the specific mechanisms and optimal application protocols of various Se sources and dosages in relation to broiler growth performance.

### 4.2. Serum Biochemical Parameters

Serum biochemical indicators reflect changes in animal organ functions. In this study, we demonstrated that dietary supplementation with 0.30 mg/kg nano-Se in broilers improved key biochemical indicators of protein and lipid metabolism, including TP, ALB, BUN, and LPS activity. Serum TP and BUN concentrations are commonly used indicators of protein absorption and synthesis [29]. Generally, high serum TP and low BUN concentrations usually indicate increased protein synthesis and improved nitrogen utilization efficiency [30]. The increased levels of TP, ALB, and BUN at 40 days suggest that nano-Se may actively participate in the protein metabolism of broilers. Zhang et al. [31] reported that albumin plays a crucial role in improving immunity and maintaining acid-base balance. These results suggest that nano-Se may affect protein metabolism pathways and improve immune system function.

Furthermore, the observed increase in LPS activity suggests that nano-Se may play a regulatory role in lipid metabolism. LPS is essential for lipid breakdown and its increased activity indicates a potential enhancement in fat digestion and utilization [32]. The differential effects observed in lipid metabolism across our experiments (i.e., experiment 1 and experiment 2) could stem from variations in Se sources and doses. While our study suggests that nano-Se modulates lipid metabolism, Khan et al. [33] reported no significant effects of 0.30 mg/kg SS or organic Se on serum lipid indicators. This discrepancy might be due to differences in the chemical form of Se used, as well as its bioavailability [11]. In conclusion, our study presents evidence supporting the positive influence of nano-Se on both protein and lipid metabolism in broilers. These findings provide a foundation for future research on Se supplementation strategies, particularly those involving novel nano-formulations, to optimize broiler growth and health.

### 4.3. Serum Antioxidant Status

It is well known that MDA, T-AOC, GSH-Px and GSH are important indicators of the antioxidant system [14]. Antioxidant enzyme activity indirectly reflects the ability to scavenge reactive oxygen species [7,34]. This study reinforces the role of nano-Se in enhancing the antioxidant defense system of broilers, as evidenced by changes in key biochemical markers. Based on fitted quadratic regression analyses for antioxidant parameters (GSH-Px, T-AOC, GSH, and MDA) at 21 days, the optimal dietary Se level was estimated to range from 0.20 to 0.22 mg/kg. This supports the conclusion that supplementation within this range is most effective for improving antioxidant capacity in broilers.

As GSH-Px is a Se-containing enzyme, its activity is directly tied to the Se status of the body, reflecting how nano-Se supplementation efficiently bolsters the antioxidant system [5]. Additionally, T-AOC and GSH levels, which were elevated in the nano-Se group, further support the positive influence of nano-Se on oxidative stress management. The reduction in MDA levels, a product of lipid peroxidation, suggests that nano-Se supplementation mitigates lipid oxidative damage, thus improving cellular integrity and overall health [14,34]. These findings are consistent with those of El-Deep et al. [35], who demonstrated that nano-Se reduced MDA levels and enhanced GSH-Px activity under high-temperature conditions. This supports the hypothesis that nano-Se not only improves antioxidant capacity but also helps broilers better cope with environmental stressors by reducing oxidative damage.

The observed enhancement in antioxidant enzyme activity, particularly with nano-Se and SY, compared to SS, which may be attributed to different Se sources have varying bioavailability [11]. Bakhshalinejad et al. [7] reported similar findings, where nano-Se and SY outperformed SS in reducing MDA levels and improving antioxidant capacity in broiler muscle and liver. Moreover, our findings align with previous studies on local Chinese Subei chickens, which showed that dietary supplementation with SY or nano-Se improved antioxidant capacity more effectively than SS at the same dosage [27]. This consistent pattern suggests that both nano-Se and SY provide a more potent antioxidant boost, likely due to their enhanced capacity to be absorbed and utilized in metabolic processes compared to SS.

### 4.4. Carcass Traits

Carcass traits are vital economic indicators in poultry production, reflecting both carcass composition and meat production performance. In this study, dietary supplementation with 0.20 mg/kg nano-Se significantly improved carcass yield, with quadratic regression analysis converging on this same dosage as optimal for carcass development. However, no significant effect on half-eviscerated carcass and eviscerated carcass yields was noted, suggesting that the increase in carcass yield may be attributed to an improvement in organ mass or other specific tissue growth, even though organ indices were not measured in this trial. This hypothesis is consistent with previous findings by Baowei et al. [34], who reported that dietary SY significantly improved immune organ indices in geese. Further studies are necessary to clarify the effect of nanosized Se on organ indices.

In terms of carcass composition, our findings are in line with the results of Khan et al. [33], who found that organic Se increased breast yield, thigh yield, and overall carcass yield in naked neck chickens compared to SS. Both nano-Se and SY were shown to enhance carcass traits more effectively than SS in our study, indicating that the bioavailability of Se sources plays a critical role in carcass development. Nano-Se and SY likely facilitate better nutrient absorption and tissue development, possibly through enhanced selenoprotein synthesis, which is crucial for growth and metabolic functions in broilers [5]. However, it is important to note the inconsistencies in the literature regarding the effects of Se supplementation on carcass traits. Bakhshalinejad et al. [1] found no significant differences in breast yield, thigh yield, or carcass yield among broilers fed diets supplemented with 0.10 mg/kg or 0.30 mg/kg SS, nano-Se, or SY. These discrepancies may stem from variations in experimental conditions, such as differences in the Se sources and their bioavailability, the duration and dosage of supplementation.

### 4.5. Meat Quality

Meat color is a critical sensory attribute influencing consumer purchase decisions, with redness being particularly important. In this study, dietary supplementation with nano-Se was found to increase the redness values of broiler muscle, consistent with the findings of Hou et al. [36], who observed that Se-enriched Saccharomyces cerevisiae increased the redness of broiler muscle. The primary contributor to meat redness is myoglobin, a heme protein responsible for oxygen storage in muscle [37]. Myoglobin oxidizes and turns brown over time, reducing meat color appeal [37,38]. Se, as an antioxidant, can enhance muscle myoglobin stability, preventing its oxidation and maintaining a bright red color [39]. Faustman et al. [38] noted that enhanced antioxidant capacity in muscle tissues can delay myoglobin oxidation and preserve meat color.

Additionally, our study showed that both nano-Se and SY supplementation reduced drip loss and cooking loss in broiler muscle compared to the SS. This finding corroborates previous research [1,27,28], which demonstrated that nano-Se and organic Se forms are superior to SS in improving muscle water-holding capacity. The water-holding capacity of muscle is closely tied to its structural integrity and is negatively impacted by the oxidation of fats and proteins. Oxidative damage weakens muscle cell membranes, causing more water to escape during storage and cooking. By improving antioxidant capacity, Se helps preserve cellular integrity, reducing water loss and enhancing meat juiciness and tenderness [2,25,40].

The strong correlation between antioxidant capacity and meat quality has been widely reported in the literature [41,42], and our results further support this relationship. Broilers receiving nano-Se or SY in their diet not only showed improved antioxidant capacity, as indicated by higher GSH-Px and lower MDA levels, but also exhibited better meat quality traits such as reduced drip and cooking loss. This suggests that the enhanced antioxidant capacity contributes to maintaining cellular integrity, thereby improving overall meat quality.

### 4.6. Tissue Se Content

The results of experiment 1 demonstrated a significant effect of nano-Se supplementation on the Se content in both the serum and breast muscle. Nano-Se supplementation exhibited a linear and quadratic relationship with Se content in the serum (linear, *p* < 0.001; quadratic, *p* = 0.077) and breast muscle (linear, *p* = 0.005; quadratic, *p* = 0.003), suggesting a dose-dependent response. These findings are consistent with those of Wang et al. [25], who reported that Se nanoparticles at levels of 0.20 and 0.50 mg/kg significantly increased Se content in the liver and muscle of broiler chickens. Likewise, Cai et al. [2] observed a dose-dependent accumulation of Se in the liver and muscle with nano-Se supplementation, while Zhou and Wang [40] also reported a similar dose–response relationship between dietary Se levels and tissue Se deposition.

The increase in tissue Se content can be explained by the higher bioavailability of nano-Se compared to other Se sources. Nano-Se, due to its smaller particle size, has a larger surface area, allowing for enhanced absorption and utilization within the body [11]. Once absorbed, Se is primarily metabolized by the liver, where it is either incorporated into selenoproteins or excreted as Se metabolites [43]. The enhanced bioavailability of nano-Se likely promotes more efficient selenoprotein synthesis, which is crucial for various metabolic and antioxidant functions, while also supporting a higher tolerance for Se accumulation [10]. These findings highlight the potential advantages of using nano-Se as a Se supplement in broilers to optimize the Se content in tissues. Despite the observed increase in tissue Se content, the selenium levels in muscle are unlikely to exceed the tolerable upper intake level for consumers, particularly for sensitive populations such as toddlers, suggesting that the risk of excessive selenium intake from poultry products is low.

The source of Se plays a critical role in Se deposition in tissues. Results from Experiment 2 showed that all Se sources (nano-Se, SS, and SY) significantly increased serum Se levels compared to the control group. However, only nano-Se and SY significantly increased muscle Se content compared to the SS group. This finding aligns with the research of Bakhshalinejad et al. [1] and Ibrahim et al. [28], which demonstrated that nano-Se and SY are retained more effectively in the body compared to SS. The differences in tissue Se deposition are likely due to variations in the absorption mechanisms of different Se sources. SS is believed to be passively absorbed, whereas SY, being an organic Se source, is absorbed through active transport mechanisms, leading to greater tissue retention [43]. Although nano-Se has consistently shown better tissue retention and bioavailability compared to SS [1,25,28,44], the exact metabolic pathways involved in nano-Se absorption and utilization remain poorly understood.

## 5. Conclusions

This study recommends 0.20–0.22 mg/kg as the optimal nano-Se supplementation for broilers, based on quadratic regression analyses of FCR (days 1–21), carcass traits, and antioxidant parameters (GSH-Px, T-AOC, GSH, and MDA levels) at day 21. At 0.20 mg/kg, nano-Se enhances antioxidant capacity, tissue Se deposition, and carcass yield. Nano-Se exhibits bioefficacy comparable to SY and superior to SS, particularly in improving feed intake and antioxidant status. These findings offer practical guidance for improving selenium supplementation strategies in broiler production.

## Figures and Tables

**Table 1:** Ingredient composition and nutrient levels of the basal diets (%, as-fed basis).

Ingredients	Starter (1–21 Days)	Grower (22–40 Days)
Corn	50.40	55.40
Soybean meal, 43%	35.40	26.80
Corn gluten meal	5.00	5.00
Flour	2.00	4.00
Soybean oil	2.80	4.50
Calcium hydrophosphate	1.80	1.35
Limestone	1.20	1.50
Salt	0.35	0.30
Vitamin premix ^1^	0.03	0.02
Trace element premix ^2^	0.15	0.15
Choline chloride, 50%	0.20	0.15
DL-Methionine	0.26	0.25
Antioxidant	0.02	0.02
L-Lysine hydrochloride	0.36	0.43
Threonine	0.00	0.10
Phytase	0.03	0.03
Total	100.00	100.00
Calculated nutrient levels ^3^		
Apparent metabolizable energy, kcal/kg	2998.00	3160.00
Crude protein	21.89	19.70
Lysine	1.30	1.15
Methionine	0.58	0.54
Methionine + cysteine	0.93	0.86
Threonine	0.85	0.72
Tryptophan	0.26	0.23
Calcium	0.95	0.94
Non-phytate phosphorus	0.40	0.32

^1^ The trace mineral premix provided the following per kg of diets: Cu, 16 mg (as CuSO_4_·5H_2_O); Zn, 110 mg (as ZnSO_4_); Fe, 80 mg (as FeSO_4_·H_2_O); Mn, 120 mg (as MnSO_4_·H_2_O); I, 1.5 mg (as CaIO_3_); Co, 0.5 mg (CoCl_2_·H_2_O). ^2^ The vitamin premix provided the following per kg of diet: vitamin A, 10,000 IU; vitamin D3, 3600 IU; vitamin E, 20 mg; vitamin K3, 2 mg; vitamin B1, 2 mg; vitamin B2, 6.4 mg; VB6, 3 mg; VB12, 0.02 mg; biotin, 0.1 mg; folic acid, 1 mg; pantothenic acid, 10 mg; nicotinamide, 30 mg. ^3^ Nutrient levels were calculated values.

**Table 2:** Analyzed selenium (Se) concentrations in experimental diets.

Treatments	Added Se (mg/kg) ^1^	Analyzed Se Concentrations (mg/kg)	
		Starter Diets	Grower Diets
Con	0	0.10	0.08
0.10 mg/kg nano-Se	0.10	0.19	0.14
0.20 mg/kg nano-Se	0.20	0.25	0.25
0.30 mg/kg nano-Se	0.30	0.37	0.37
0.40 mg/kg nano-Se	0.40	0.43	0.44
SS	0.20	0.32	0.36
SY1	0.20	0.13	0.29
SY2	0.20	0.23	0.26

Abbreviations: Con, control group; nano-Se, nano-selenium group; SY1, Se-enriched yeast from Angel Yeast Co; SY2, Se-enriched yeast from Lallemend Co. ^1^ All Se levels were expressed on an elemental Se basis (mg/kg diet), and inclusion rates of different Se sources were adjusted according to their Se content.

**Table 3:** Effect of nano-Se supplementation on growth performance of broilers.

Items	Nano-Se, mg/kg					SEM	*p*-Value		
	0	0.10	0.20	0.30	0.40		ANOVA	Linear	Quadratic
d 1–21									
d 21 BW, g	887.62	863.59	868.03	882.69	870.41	5.198	0.571	0.686	0.454
ADFI, g/d	50.91	49.37	49.36	50.75	51.04	0.308	0.196	0.462	0.059
ADG, g/d	40.11	38.96	39.16	39.87	39.28	0.248	0.561	0.678	0.449
FCR	1.28	1.27	1.26	1.27	1.28	0.003	0.266	0.848	0.044
Mortality rate, %	2.08	1.04	0.00	0.00	0.00	0.348	0.222	0.038	0.277
d 22–40									
d 40 BW, g	2381.13	2347.91	2396.81	2474.08	2408.42	17.513	0.231	0.144	0.799
ADFI, g/d	129.09	129.95	134.47	136.66	132.87	1.049	0.118	0.050	0.169
ADG, g/d	78.61	78.12	80.32	84.55	80.95	0.792	0.082	0.044	0.517
FCR	1.64	1.64	1.65	1.63	1.66	0.006	0.630	0.522	0.392
Mortality rate, %	0.00	0.00	0.00	0.00	1.11	0.222	0.426	0.170	0.243
d 1–40									
ADFI, g/d	87.92	87.53	89.92	91.46	90.30	0.606	0.189	0.041	0.913
ADG, g/d	58.40	57.56	58.57	60.72	59.07	0.458	0.255	0.158	0.873
FCR	1.54	1.56	1.55	1.54	1.57	0.005	0.406	0.415	0.510
Mortality rate, %	2.08	1.04	0.00	0.00	1.04	0.395	0.440	0.274	0.128

Abbreviations: BW, body weight; ADFI, average daily feed intake; ADG, average daily gain; FCR, feed conversion ratio.

**Table 4:** Effect of nano-Se supplementation on serum biochemical parameters of broilers.

Items	Nano-Se, mg/kg					SEM	*p*-Value		
	0	0.10	0.20	0.30	0.40		ANOVA	Linear	Quadratic
21 d									
TP, g/L	26.79	28.99	29.92	29.12	29.28	0.387	0.135	0.076	0.080
ALB, g/L	17.20	17.39	19.58	18.32	18.70	0.357	0.158	0.097	0.266
GLB, g/L	10.09	11.6	10.59	9.87	9.73	0.263	0.134	0.182	0.140
TC, mmol/L	3.10	3.29	3.50	3.44	3.48	0.054	0.100	0.018	0.193
TG, mmol/L	0.38 ^b^	0.36 ^b^	0.76 ^a^	0.39 ^b^	0.48 ^b^	0.035	0.001	0.195	0.066
HDL, mmol/L	1.61	1.70	1.83	1.78	1.84	0.031	0.090	0.013	0.343
LDL, mmol/L	1.42	1.52	1.51	1.55	1.55	0.023	0.340	0.085	0.354
VLDL, mmol/L	2.80	3.12	2.98	3.23	3.10	0.061	0.212	0.094	0.307
FFA, umol/L	342.58	349.63	336.24	325.54	322.19	7.388	0.775	0.244	0.781
BUN, mmol/L	1.57	2.00	1.98	1.75	1.74	0.060	0.112	0.797	0.027
LPS, U/L	9.78 ^b^	11.94 ^a^	11.32 ^a^	12.12 ^a^	11.81 ^a^	0.196	<0.001	<0.001	0.002
40 d									
TP, g/L	30.14 ^b^	29.67 ^b^	28.57 ^b^	32.13 ^a^	30.20 ^b^	0.326	0.004	0.149	0.459
ALB, g/L	17.17 ^b^	17.57 ^b^	17.62 ^b^	20.30 ^a^	18.87 ^ab^	0.354	0.030	0.013	0.737
GLB, g/L	12.96	12.10	11.34	11.14	11.34	0.282	0.246	0.045	0.261
TC, mmol/L	3.11 ^b^	3.04 ^bc^	2.80 ^c^	3.46 ^a^	3.10 ^b^	0.053	0.002	0.267	0.284
TG, mmol/L	0.20 ^c^	0.20 ^c^	0.20 ^c^	0.78 ^a^	0.41 ^b^	0.050	<0.001	<0.001	0.346
HDL, mmol/L	1.86	1.88	1.69	1.61	1.64	0.040	0.081	0.012	0.660
LDL, mmol/L	1.10 ^c^	1.12 ^c^	1.10 ^c^	1.76 ^a^	1.38 ^b^	0.058	<0.001	<0.001	0.487
VLDL, mmol/L	2.66 ^c^	2.46 ^c^	2.49 ^c^	3.38 ^a^	2.94 ^b^	0.073	<0.001	<0.001	0.307
FFA, umol/L	357.70 ^ab^	322.54 ^b^	339.29 ^b^	436.05 ^a^	391.73 ^ab^	12.998	0.028	0.034	0.693
BUN, mmol/L	1.17 ^b^	1.28 ^ab^	1.07 ^b^	1.50 ^a^	1.33 ^ab^	0.046	0.021	0.069	0.806
LPS, U/L	12.50 ^c^	11.53 ^c^	11.89 ^c^	16.24 ^a^	14.16 ^b^	0.369	<0.001	<0.001	0.296

Abbreviations: TP, total protein; ALB, albumin; GLB, globulin; TC, total cholesterol; TG, triglycerides; HDL, high density lipoprotein; LDL, low density lipoprotein; VLDL, very-low-density lipoprotein; FFA, free fatty acids; BUN, blood urea nitrogen; LPS, lipase. ^a, b, c^ Means that do not share similar letter in row are significantly different, *p* < 0.05.

**Table 5:** Effect of nano-Se supplementation on serum antioxidant parameters of broilers.

Items	Nano-Se, mg/kg					SEM	*p*-Value		
	0	0.10	0.20	0.30	0.40		ANOVA	Linear	Quadratic
21 d									
MDA, nmol/mL	6.10 ^a^	5.36 ^ab^	4.65 ^b^	4.58 ^b^	5.93 ^a^	0.165	0.001	0.220	<0.001
T-AOC, U/mL	10.92	11.22	11.74	11.67	11.01	0.113	0.064	0.463	0.008
GSH-Px, U/mL	881.41 ^b^	984.88 ^ab^	1036.18 ^a^	1035.03 ^a^	907.97 ^ab^	15.818	<0.001	0.205	<0.001
GSH, U/mL	6.97 ^b^	7.77 ^a^	8.47 ^a^	8.6 ^a^	6.95 ^b^	0.169	<0.001	0.334	<0.001
40 d									
MDA, nmol/mL	5.19 ^bc^	5.94 ^a^	4.84 ^c^	5.34 ^bc^	5.52 ^ab^	0.103	0.002	0.820	0.489
T-AOC, U/mL	11.12 ^abc^	10.71 ^bc^	11.65 ^a^	11.27 ^ab^	10.62 ^c^	0.112	0.006	0.483	0.016
GSH-Px, U/mL	959.66 ^ab^	886.05 ^c^	1012.31 ^a^	964.81 ^ab^	928.64 ^bc^	12.619	0.007	0.820	0.489
GSH, U/mL	7.79 ^ab^	7.11 ^bc^	8.27 ^a^	7.42 ^bc^	6.86 ^c^	0.135	0.001	0.046	0.020

Abbreviations: MDA, malondialdehyde; T-AOC, total antioxidant capacity; GSH-Px, Glutathione peroxidase; GSH, reduced glutathione. ^a, b, c^ Means that do not share similar letter in row are significantly different, *p* < 0.05.

**Table 6:** Effect of nano-Se supplementation on carcass trait of broilers.

Items	Nano-Se, mg/kg					SEM	*p*-Value		
	0	0.10	0.20	0.30	0.40		ANOVA	Linear	Quadratic
Carcass yield, %	92.69 ^b^	93.40 ^b^	94.88 ^a^	93.61 ^ab^	92.55 ^b^	0.250	0.025	0.739	0.003
Half-eviscerated carcass, %	86.76	87.11	88.42	86.49	86.90	0.285	0.306	0.960	0.212
Eviscerated carcass, %	75.17	75.69	75.99	75.52	74.28	0.254	0.340	0.377	0.063
Breast muscle yield, %	26.78	27.69	27.45	28.22	30.13	0.503	0.288	0.049	0.509
Thigh muscle yield, %	19.93	20.30	19.30	20.51	21.18	0.284	0.316	0.110	0.416
Abdominal fat yield, %	1.59	1.61	1.86	1.73	1.45	0.070	0.549	0.921	0.164

^a, b^ Means that do not share similar letter in row are significantly different, *p* < 0.05.

**Table 7:** Effect of nano-Se supplementation on meat quality of broilers.

Items	Nano-Se, mg/kg					SEM	*p*-Value		
	0	0.10	0.20	0.30	0.40		ANOVA	Linear	Quadratic
pH_45min_	7.07	7.02	7.05	7.14	7.17	0.037	0.574	0.108	0.806
pH_24h_	5.98	6.07	6.00	6.13	5.91	0.027	0.089	0.725	0.062
Drip loss, %	2.17	2.32	2.39	2.24	2.18	0.039	0.360	0.629	0.075
Cooking loss, %	24.95	25.14	23.83	24.96	21.84	0.515	0.237	0.090	0.359
Shear force, N	28.71	32.00	37.09	27.16	24.69	2.067	0.364	0.343	0.138
Color values at 45 min postmortem									
Lightness, L_45min_	41.98	42.21	41.52	41.61	41.32	0.296	0.899	0.397	0.921
Redness, a_45min_	6.99 ^c^	8.31 ^ab^	7.74 ^bc^	7.71 ^bc^	9.15 ^a^	0.211	0.010	0.006	0.600
Yellowness, b_45min_	9.56 ^abc^	9.93 ^ab^	8.52 ^c^	9.30 ^bc^	10.45 ^a^	0.195	0.013	0.243	0.011
Color values at 24 h postmortem									
Lightness, L_24h_	41.81	43.53	43.36	42.07	42.95	0.452	0.706	0.808	0.482
Redness, a_24h_	3.52	4.07	3.70	3.83	4.25	0.106	0.199	0.094	0.713
Yellowness, b_24h_	9.06	9.87	9.07	9.51	9.61	0.204	0.712	0.581	0.901

^a, b, c^ Means that do not share similar letter in row are significantly different, *p* < 0.05.

**Table 8:** Effect of nano-Se supplementation on tissue selenium content of broilers.

Items	Nano-Se, mg/kg					SEM	*p*-Value		
	0	0.10	0.20	0.30	0.40		ANOVA	Linear	Quadratic
Serum Se, ug/mL	0.06 ^b^	0.16 ^a^	0.18 ^a^	0.19 ^a^	0.21 ^a^	0.015	0.001	<0.001	0.077
Breast Se, ug/g	0.08	0.13	0.18	0.18	0.20	0.014	0.152	0.020	0.372

^a, b^ Means that do not share similar letter in row are significantly different, *p* < 0.05.

**Table 9:** Regression models for estimating Se requirements in broilers.

Dependent Variables ^1^	Regression Equation ^2^	R^2^	Se Requirement, mg/kg
FCR from 1 to 21 d	y = 0.4286x^2^ − 0.1714x + 1.2806	0.9184	0.20
Carcass yield, %	y = −44.929x^2^ + 17.901x + 92.541	0.8174	0.20
Yellowness, b_45min_	y = 26.786x^2^ − 9.5643x + 9.8577	0.5471	0.18
MDA at 21 d, nmol/mL	y = 34.429x^2^ − 14.891x + 6.2366	0.9021	0.22
T-AOC at 21 d, U/mL	y = −17.929x^2^ + 7.8014x + 10.827	0.8672	0.22
GSH-Px at 21 d, U/mL	y = −3667.9x^2^ + 1570.4x + 875.08	0.9698	0.21
GSH at 21 d, U/mL	y = −39.071x^2^ + 16.419x + 6.8126	0.8835	0.21
T-AOC at 40 d, U/mL	y = −12.857x^2^ + 4.7029x + 10.905	0.3528	0.18
GSH at 40 d, U/mL	y = −12.643x^2^ + 3.5071x + 7.5471	0.3728	0.14

Abbreviations: FCR, feed conversion ratio; MDA, malondialdehyde; GSH-Px, glutathione peroxidase; GSH, reduced glutathione; T-AOC, total antioxidant capacity. ^1^ All variables presented had significant quadratic contrasts. ^2^ Y-variable = response outcome and X-variable = calculated dietary selenium level.

**Table 10:** Effects of supplemental selenium source on growth performance of broilers.

Items	Con	Nano-Se	SS	SY1	SY2	SEM	*p*-Value
d 1–21							
d 21 BW, g	887.62	868.03	869.97	867.67	890.15	5.402	0.512
ADFI, g/d	50.91	49.36	50.81	50.18	51.19	0.271	0.213
ADG, g/d	40.11	39.16	39.25	39.14	40.22	0.258	0.501
FCR	1.28	1.26	1.28	1.28	1.28	0.003	0.106
Mortality rate, %	2.08	0.00	0.00	0.00	1.04	0.348	0.222
d 22–40							
d 40 BW, g	2381.13	2396.81	2302.24	2368.44	2446.23	93.749	0.105
ADFI, g/d	129.09 ^ab^	134.47 ^a^	123.74 ^b^	131.10 ^ab^	136.19 ^a^	7.264	0.016
ADG, g/d	78.61	80.32	75.44	79.39	81.90	4.493	0.140
FCR	1.64	1.65	1.64	1.65	1.68	0.042	0.610
Mortality rate, %	0.00	0.00	0.00	1.11	1.11	0.309	0.567
d 1–40							
ADFI, g/d	87.92 ^ab^	88.92 ^ab^	84.81 ^b^	89.79 ^a^	91.97 ^a^	0.736	0.029
ADG, g/d	58.40	58.57	56.41	58.80	60.90	0.468	0.061
FCR	1.54	1.55	1.54	1.57	1.57	0.006	0.344
Mortality rate, %	2.08	0.00	0.00	1.04	1.04	0.407	0.481

Abbreviations: BW, body weight; ADFI, average daily feed intake; ADG, average daily gain; FCR, feed conversion ratio; Con, control group; nano-Se, nano-selenium group; SY1, Se-enriched yeast from Angel Yeast Co; SY2, Se-enriched yeast from Lallemend Co. ^a, b^ Means that do not share similar letter in row are significantly different, *p* < 0.05.

**Table 11:** Effects of supplemental selenium source on serum biochemical parameters of broilers.

Items	Con	Nano-Se	SS	SY1	SY2	SEM	*p*-Value
21 d							
TP, g/L	26.79 ^c^	29.92 ^ab^	31.38 ^a^	29.34 ^b^	28.95 ^b^	0.388	0.001
ALB, g/L	17.20 ^b^	19.58 ^a^	20.14 ^a^	19.63 ^a^	19.12 ^a^	0.256	<0.001
GLB, g/L	10.09 ^b^	10.59 ^ab^	12.03 ^a^	10.40 ^ab^	9.21 ^b^	0.298	0.036
TC, mmol/L	3.10 ^b^	3.50 ^a^	3.44 ^a^	3.76 ^a^	3.56 ^a^	0.061	0.007
TG, mmol/L	0.38 ^b^	0.76 ^a^	0.50 ^b^	0.39 ^b^	0.32 ^b^	0.037	0.001
HDL, mmol/L	1.61 ^b^	1.83 ^a^	1.79 ^ab^	1.97 ^a^	1.85 ^a^	0.034	0.011
LDL, mmol/L	1.42 ^c^	1.51 ^bc^	1.59 ^ab^	1.69 ^a^	1.69 ^a^	0.029	0.001
VLDL, mmol/L	2.80 ^c^	2.98 ^bc^	3.17 ^abc^	3.43 ^a^	3.34 ^ab^	0.067	0.009
FFA, umol/L	342.58	336.24	329.13	348.85	357.83	4.987	0.432
BUN, mmol/L	1.57 ^c^	1.98 ^ab^	2.00 ^a^	1.77 ^abc^	1.69 ^bc^	0.050	0.011
LPS, U/L	9.78 ^c^	11.32 ^b^	12.22 ^ab^	13.23 ^a^	12.89 ^a^	0.273	<0.001
40 d							
TP, g/L	30.14	28.57	30.09	29.00	30.26	0.336	0.386
ALB, g/L	17.17	17.62	18.81	17.71	19.04	0.355	0.400
GLB, g/L	12.96 ^a^	11.34 ^b^	11.28 ^b^	10.96 ^b^	11.22 ^b^	0.234	0.049
TC, mmol/L	3.11 ^a^	2.80 ^b^	3.17 ^a^	3.07 ^ab^	3.29 ^a^	0.052	0.033
TG, mmol/L	0.20 ^b^	0.20 ^b^	0.30 ^b^	0.22 ^b^	0.53 ^a^	0.039	0.011
HDL, mmol/L	1.86	1.69	1.85	1.83	1.73	0.035	0.416
LDL, mmol/L	1.10 ^b^	1.10 ^b^	1.26 ^ab^	1.13 ^b^	1.46 ^a^	0.041	0.006
VLDL, mmol/L	2.66 ^b^	2.49 ^b^	2.68 ^b^	2.58 ^b^	2.97 ^a^	0.047	0.007
FFA, umol/L	357.70	339.29	368.87	336.42	404.86	11.333	0.284
BUN, mmol/L	1.17 ^b^	1.07 ^b^	1.03 ^b^	1.14 ^b^	1.47 ^a^	0.049	0.017
LPS, U/L	12.50 ^b^	11.89 ^b^	12.56 ^b^	11.51 ^b^	14.27 ^a^	0.235	<0.001

Abbreviations: TP, total protein; ALB, albumin; GLB, globulin; TC, total cholesterol; TG, triglycerides; HDL, high density lipoprotein; LDL, low density lipoprotein; VLDL, very-low-density lipoprotein; FFA, free fatty acids; BUN, blood urea nitrogen; LPS, lipase. Con, control group; nano-Se, nano-selenium group; SY1, Se-enriched yeast from Angel Yeast Co; SY2, Se-enriched yeast from Lallemend Co. ^a, b, c^ Means that do not share similar letter in row are significantly different, *p* < 0.05.

**Table 12:** Effects of supplemental selenium source on serum antioxidant parameters of broilers.

Items	Con	Nano-Se	SS	SY1	SY2	SEM	*p*-Value
21 d							
MDA, nmol/mL	6.10 ^a^	4.65 ^b^	5.91 ^a^	3.98 ^b^	4.26 ^b^	0.187	<0.001
T-AOC, U/mL	10.92 ^c^	11.74 ^b^	10.84 ^c^	12.26 ^a^	12.10 ^ab^	0.134	<0.001
GSH-Px, U/mL	881.41 ^b^	1036.18 ^a^	908.91 ^b^	1079.57 ^a^	1091.35 ^a^	19.288	<0.001
GSH, U/mL	6.97 ^b^	8.47 ^a^	7.20 ^b^	8.99 ^a^	8.53 ^a^	0.169	<0.001
40 d							
MDA, nmol/mL	6.10 ^a^	4.65 ^b^	5.76 ^a^	3.98 ^b^	4.26 ^b^	0.180	<0.001
T-AOC, U/mL	11.12 ^c^	11.65 ^bc^	11.59 ^bc^	12.39 ^a^	11.92 ^ab^	0.119	0.013
GSH-Px, U/mL	881.41 ^b^	1036.18 ^a^	908.91 ^b^	1096.06 ^a^	1091.35 ^a^	19.594	<0.001
GSH, U/mL	7.79	8.27	8.21	9.02	8.77	0.145	0.091

Abbreviations: MDA, malondialdehyde; T-AOC, total antioxidant capacity; GSH-Px, glutathione peroxidase; GSH, reduced glutathione; Con, control group; nano-Se, nano-selenium group; SY1, Se-enriched yeast from Angel Yeast Co; SY2, Se-enriched yeast from Lallemend Co. ^a, b, c^ Means that do not share similar letter in row are significantly different, *p* < 0.05.

**Table 13:** Effects of supplemental selenium source on carcass trait of broilers.

Items	Con	Nano-Se	SS	SY1	SY2	SEM	*p*-Value
Carcass yield, %	92.69 ^ab^	94.88 ^a^	89.14 ^c^	93.35 ^ab^	92.67 ^b^	0.477	0.002
Half-eviscerated carcass, %	86.76 ^a^	88.42 ^a^	82.47 ^b^	88.03 ^a^	87.63 ^a^	0.496	<0.001
Eviscerated carcass, %	75.17 ^a^	75.99 ^a^	71.69 ^b^	75.00 ^a^	75.37 ^a^	0.355	<0.001
Breast muscle yield, %	26.78	27.45	27.88	27.19	28.40	0.339	0.620
Thigh muscle yield, %	19.93	19.30	18.91	19.90	19.34	0.268	0.222
Abdominal fat yield, %	1.59 ^ab^	1.86 ^a^	1.34 ^bc^	1.76 ^ab^	1.07 ^c^	0.079	0.006

Abbreviations: Con, control group; nano-Se, nano-selenium group; SY1, Se-enriched yeast from Angel Yeast Co; SY2, Se-enriched yeast from Lallemend Co. ^a, b, c^ Means that do not share similar letter in row are significantly different, *p* < 0.05.

**Table 14:** Effects of supplemental selenium source on meat quality of broilers.

Items	Con	Nano-Se	SS	SY1	SY2	SEM	*p*-Value
pH_45min_	7.07	7.05	7.21	7.07	6.89	0.045	0.311
pH_24h_	5.98	6.00	6.00	5.99	6.07	0.018	0.499
Drip loss, %	2.17 ^b^	2.39 ^b^	3.35 ^a^	2.13 ^b^	2.39 ^b^	0.109	<0.001
Cooking loss, %	24.95 ^ab^	23.83 ^b^	27.07 ^a^	27.24 ^a^	24.13 ^b^	0.471	0.037
Shear force, N	28.71	37.09	40.12	28.87	20.17	2.507	0.074
Color values at 45 min postmortem							
Lightness, L_45min_	41.98	41.52	42.95	42.64	42.14	0.409	0.914
Redness, a_45min_	6.99 ^b^	7.74 ^ab^	7.66 ^ab^	8.02 ^a^	6.94 ^b^	0.163	0.037
Yellowness, b_45min_	9.56	8.52	9.70	9.41	9.53	0.192	0.256
Color values at 24 h postmortem							
Lightness, L_24h_	41.81	43.36	42.60	42.12	42.01	0.414	0.678
Redness, a_24h_	3.52	3.70	4.43	4.65	4.65	0.161	0.052
Yellowness, b_24h_	9.06	9.07	9.61	9.08	9.26	0.184	0.672

Abbreviations: Con, control group; nano-Se, nano-selenium group; SY1, Se-enriched yeast from Angel Yeast Co; SY2, Se-enriched yeast from Lallemend Co. ^a, b^ Means that do not share similar letter in row are significantly different, *p* < 0.05.

**Table 15:** Effects of supplemental selenium source on serum and breast selenium content of broilers.

Items	Con	Nano-Se	SS	SY1	SY2	SEM	*p*-Value
Serum Se, ug/mL	0.06 ^b^	0.18 ^a^	0.16 ^a^	0.22 ^a^	0.18 ^a^	0.015	0.001
Breast Se, ug/g	0.08 ^b^	0.18 ^a^	0.14 ^ab^	0.20 ^a^	0.20 ^a^	0.012	0.010

Abbreviations: Con, control group; nano-Se, nano-selenium group; SY1, Se-enriched yeast from Angel Yeast Co; SY2, Se-enriched yeast from Lallemend Co. ^a, b^ Means that do not share similar letter in row are significantly different, *p* < 0.05.

## Data Availability

The original contributions presented in this study are included in the article. Further inquiries can be directed to the corresponding authors.
